# Ethnobotanical survey of wild food plants traditionally collected and consumed in the Middle Agri Valley (Basilicata region, southern Italy)

**DOI:** 10.1186/s13002-017-0177-4

**Published:** 2017-09-06

**Authors:** Sabrina Sansanelli, Maura Ferri, Mirko Salinitro, Annalisa Tassoni

**Affiliations:** Department of Biological, Geological and Environmental Sciences, University of Bologna, Via Irnerio 42, 40126 Bologna, Italy

**Keywords:** Ethnobotany, Traditional local knowledge, Wild food plants, Agri Valley, Basilicata region, *Cichorium Intybus*

## Abstract

**Background:**

This research was carried out in a scarcely populated area of the Middle Agri Valley (Basilicata region, southern Italy). The aim of the study was to record local knowledge on the traditional uses of wild food plants, as well as to collect information regarding the practices (gathering, processing and cooking) and the medicinal uses related to these plants.

**Methods:**

Fifty-eight people still possessing traditional local knowledge (TLK), 74% women and 26% men, were interviewed between May–August 2012 and January 2013, using open and semi-structured ethnobotanical interviews. For each described plant species, the botanical family, the Italian common and folk names, the plant parts used, the culinary preparation and, when present, the medicinal use, were recorded and the relative frequency of citation index (RFC) was determined.

**Results:**

The 52 plant species mentioned by the respondents belong to 23 botanical families, with Asteraceae (12 plants) and Rosaceae (7 plants) being most frequently cited. The species with the highest RFC index is *Cichorium intybus* L. (0.95), followed by *Sonchus* spp. (*S. oleraceus* L., *S. asper* L. and *S. arvensis* L.) (0.76). The plant parts preferably used are leaves (22 plants), fruits (12) and stems (7). Only six wild plants were indicated as having both food use and therapeutic effect.

**Conclusions:**

The survey conducted on the traditional use of wild food plants in the Middle Agri Valley revealed that this cultural heritage is only partially retained by the population. Over the last few decades, this knowledge has been in fact quickly disappearing along with the people and, even in the rural context of the study area, is less and less handed down to younger generations. Nevertheless, data also revealed that the use of wild plants is recently being revaluated in a way closely related to local habits and traditions.

**Electronic supplementary material:**

The online version of this article (doi:10.1186/s13002-017-0177-4) contains supplementary material, which is available to authorized users.

## Background

“*Let food be thy medicine and medicine be thy food”*. This aphorism was for long time attributed to the Hippocrates (460 – about 370 BC). Although this statement never appreared as such in the so called *Hippocratic Collection* [1], it reflects the approch of the Greek physician to medicine, emphasizying for the first time the importance of diet and living habits in preventing illness and disease*.* In fact in Antiquity, a large group of plant species used for the preparation of medicines were also consumed as foods. This concept was well*-*established [2, 3] among people who traditionally gathered wild food plant species and were well aware of their health-beneficial properties. Nowadays, wild food plants are generally known to have high nutritional values, higher fibre and polyphenol contents, and greater antioxidant capacity than the corresponding cultivated species [4, 5]. Moreover, many wild greens have been demonstrated to be effective in preventing chronic diseases, such as cardiovascular pathologies and diabetes [3, 6, 7].

Over time, the gathering practices and ways of consumption of wild food plants slowly integrated into the customs of a territory, becoming part of the traditional local knowledge (TLK). More recently, the progress of industrialization, urbanization and large-scale farming has had a profound effect on society and the way of living, which became gradually less rural. As wild food plant practices and related TLK are strongly imbedded in rural societies, they have progressively disappeared.

The use of wild food plants has been investigated previously in the Mediterranean area [8, 9], in selected study sites of various countries, among which Greece, Italy, Albania, Morocco and Spain, evidencing an extremely variable use of wild plants, strongly related to traditions and cultural heritage. The results also pointed out that the habit and knowledge of using wild food plants has progressively decreased over generations [10, 11]. Changes in the contemporary use of wild food plants in Italy and other European countries have also been studied by Łuczaj and co-workers [8], confirming that the traditional use of wild edibles is rapidly decreasing due to socioeconomic and ecological changes, in particular close to urban areas. A comprehensive comparative ethnobotanical study on the use of wild food plants in Italy [9] evidenced the prevalent use of wild plants belonging to the Rosaceae family in the north, and to Asteraceae, Brassicaceae and Liliaceae (*lato* sensu) species in the south. Some botanical species, such as *Asparagus acutifolius* L., *Cichorium intybus* L. and *Foeniculum vulgare* Mill., were mentioned in several areas, while *Borago officinalis* L. was the most quoted taxon in both southern and northern Italian sites. In general, the data showed that in southern Italy the loss of wild TLK plants is happening at a slower rate than in the north [9]. Recently, a study was carried out in the province of Bologna, located in the Emilia-Romagna region (northern Italy) [12], which is one of the most economically developed regions of Italy. The informants mentioned a total of 66 wild food plants, including greens (leafy plants eaten as vegetables), fruits and semi-wild plants, which are still known and collected today. Results indicated that the popular traditions regarding wild food plants have been progressively lost, because they are not handed down anymore to new generations. Nevertheless, the survey also pointed out that the use of wild food plants in the Bologna area is recently being revaluated, mainly because they are perceived as healthier than cultivated plants and contribute to the preservation of biodiversity.

The present study was performed in the Middle Agri Valley territory located in the south-west part of the Basilicata region (southern Italy), in the province of Potenza. Several studies have been carried out before in this region but mostly in the north-central parts (especially regarding the Arbëreshë Albanian community) [13–16] and in the Tyrrhenian sector [17]. According to our knowledge, only one ethnobotanical study of the area has previously been published regarding traditional phytotherapy practices of the Agri Valley area, in which indications were given on the therapeutic uses of some cultivated and wild plants [18].

The objective of the present research was to record local knowledge concerning the traditional uses of wild food plants in the Middle Agri Valley, an area characterised by the presence of few villages, primarily sustained by agriculture, a low population density and a prevalence of old people. Collected information included gathering, processing and cooking practises and therapeutic uses of the mentioned plants, in an attempt to preserve the cultural heritage still partially retained by the investigated population.

## Methods

**Fig. 1:**
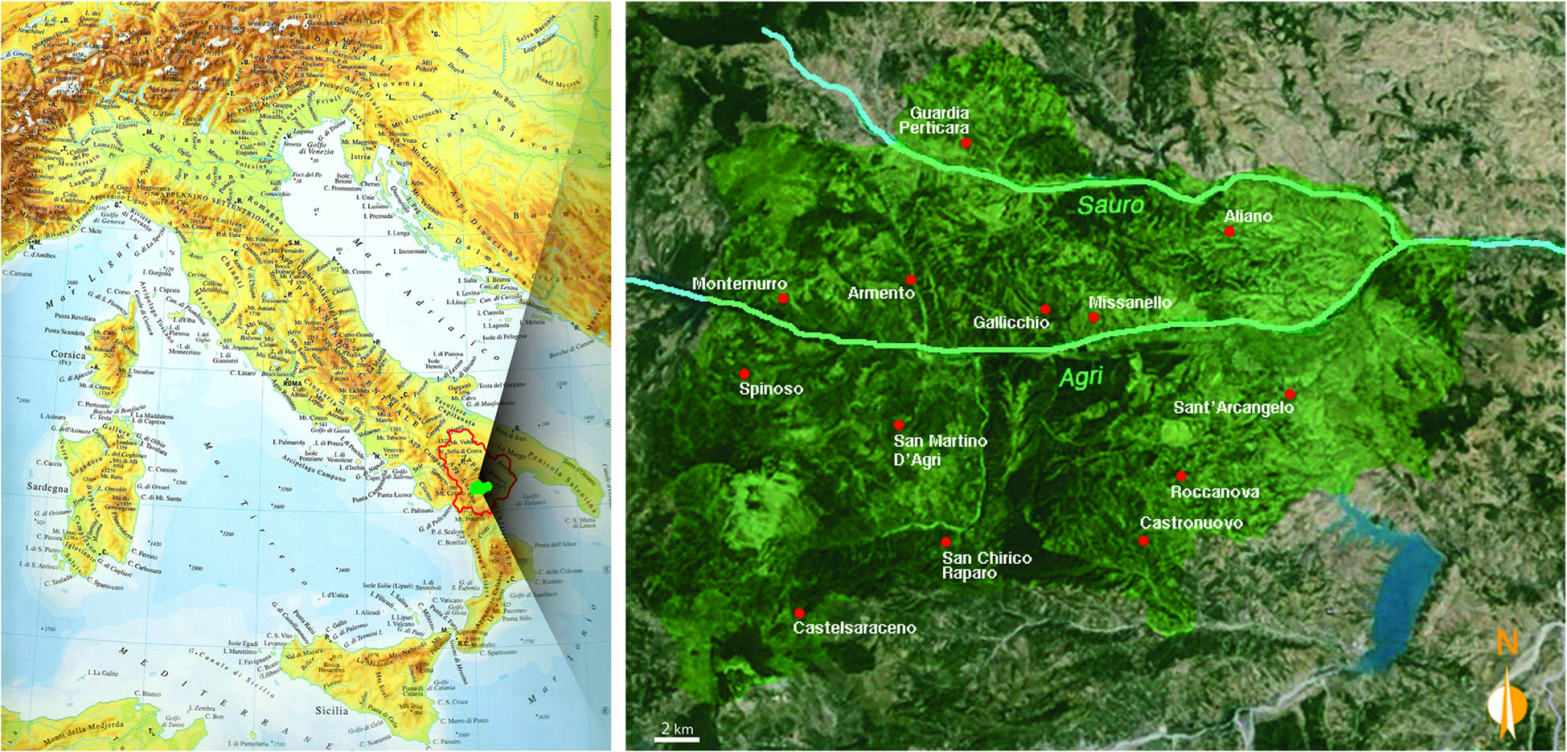
Location of the Agri Valley study area

Fieldwork was conducted in an area of the Agri Valley, located in the south-west part of Basilicata, within the province of Potenza, which takes its name from the Agri River. The valley is characterised by different environments and surrounded by the Volturino (1856 m) and Viggiano (1725 m) mountains. It may be subdivided geographically and socio-economically in three distinct parts: Upper, Middle and Lower [19]. The present survey was focused in particular on the Middle Agri Valley, which is characterised by the presence of few villages (Fig. 1), primarily sustained by agriculture. The lack of industries and its geographical isolation from commercial and tourist traffic have prompted young people leave the area and emigrate abroad or to the north of Italy. For this reason, the area has a low population density and is mainly inhabited by the elderly. The research was performed during May–August 2012 and in January 2013.

Standard ethnobotanical tools [20], such as participant observation, as well as open and semi-structured interviews, were used to collect information. Additional file 1 shows the questionnaire form used as a guideline for the interviews. The respondents were selected by snow ball sampling and a total of 58 people still retaining traditional local knowledge (TLK) were interviewed. All participants as well as their parents were born and had lived their whole life in the study area (Fig. 1). The origin of the family home is very important as TLK is formed and preserved within the family, where it is usually vertically transmitted from older to younger generations. The purpose, method, and nature of the research were explained before interviews took place and informed consent was obtained from all informants. Initially, informants were asked to freely recall all the wild plant species that they had used in the past and/or were presently using for food purposes. For each plant species mentioned, the informants were asked to provide the local name, the plant parts used, the period of harvesting, the culinary, medicinal, and other possible uses, the frequency of use and whether they had used the plant in the past and/or were still using it. Further questions regarded processing and cooking activities, therapeutic effects after consumption of particular wild food plants, and medicinal use of plants, including detailed modes of preparation and application. The perception of wild species compared to their cultivated analogues was investigated as well as their possible impacts, benefits or risks on human nutrition and health. The taste and level of appreciation of the consumed plant species were also described.

The relative frequency of citation index (RFC), i.e. the number of informants who cited a specific wild food plant divided by the total number of informants, was calculated for each mentioned plant species. Its value, which may vary from 0, when nobody refers to the plant as useful, to 1, when all informants mention the use of a species, was used as a measure for the local importance of each species [21]. The two most knowledgeable and observant informants were selected as *key informants* to become involved in participant observations. The information they provided was crucial to better understand the way of plant collection, food preparation, gender relation and mode of passing down local knowledge. The *key informants* also contributed to the gathering of the mentioned wild food plant species, which they called by the relative folk or Italian common names. Expert botanists (Dr. Mossetti Umberto and Dr. Managlia Annalisa of the University of Bologna) identified and renamed the collected wild plant specimens, following standard botanical nomenclature [22]. Voucher specimens were collected and deposited in the Herbarium of the University of Bologna (acronym BOLO).

## Results and discussion

### Informants

Fifty-eight people still holding traditional local knowledge were interviewed: 43 women (74%) and 15 men (26%), ranging in age between 33 and 96 years, with a mean of 70 and a median of 73. In general, women were more available than men to speak about wild food plants, probably because they outnumbered them among the elderly [23]. The results showed that the traditional local knowledge (TLK) memory was better preserved in women, who gave much more details and information on the used traditional wild food plants than men. This may be explained by the fact that, for many years in the past, the men of the Agri Valley, head of families, used to emigrate elsewhere for work, while women remained in the villages taking care of the family. Consequently, the gathering, processing and cooking of wild plants were almost exclusively done by women. Several other studies performed in southern Italy [24–26] also showed that women are the major depositaries of wild plant local knowledge.

### Wild food plant data

Interviewed informants cited 52 wild plant species, including greens (leafy plants eaten as vegetables), fruits and semi-wild plants, listed in Table 1 together with the relative frequency of citation (RFC) index, botanical family, plant parts used, Italian common and folk names, and the culinary and medicinal uses. The mean number of quoted species was almost equal between the two genders: 10.5 for women and 10.9 for men.

**Table 1:** List of wild food plants used in the study area of the Middle Agri Valley

Botanical name	RFC	Botanical family	Part of the plant used	Italian folk name	Culinary use	Medicinal use (preparation and administration)
*Allium schoenoprasum* L.	0.03	Amaryllidaceae	leaves		flavouring	
*Arbutus unedo* L.	0.05	Ericaceae	fruits	gan’l	fresh fruits	
*Armoracia rusticana* Gaertn., Mey. et Scherb.	0.68	Brassicaceae	roots	rafnt	flavouring, omelettes or with eggs, cheese and pasta	
*Apium nodiflorum* (L.) Lag.	0.02	Apiaceae	leaves	crescione	mixed vegetables soup, pan-fried	
*Asparagus acutifolius* L.	0.48	Asparagaceae	shoots	sparasc’	fried with eggs and salami	kidney wellbeing (cold cooking water)
*Beta vulgaris* L. ssp. *maritima* (L.) Arcang.	0.52	Amaranthaceae	leaves	iet’	alone in soup or with beans, in “stuffed pizza”	
*Borago officinalis* L.	0.43	Boraginaceae	leaves	burraccia	alone in soup or with beans, pancakes	
*Cardus pycnocephalus* L.	0.05	Asteraceae	stems	cardone, scardunecch’	boiled, pan-fried	
*Carthamus caeruleus* L.	0.03	Asteraceae	stems	cardone, scardunecch’	boiled	
*Capparis spinosa* L.	0.14	Capparaceae	buds		flavouring	
*Cichorium intybus* L.	0.95	Asteraceae	leaves	cicoria	salads, alone in soup or with beans or with mixed vegetables	liver wellbeing (cold cooking water and cooked leaves)
*Clematis vitalba* L.	0.34	Ranuncolaceae	shoots	grambullin’, vitacchia	omelettes, pan-fried with garlic, eggs and salami	
*Cirsium arvense* (L.) Scop.	0.03	Asteraceae	stems	cardone, scardunecch’	boiled	
*Cornus mas* L.	0.05	Cornaceae	fruits	curnal’	fresh fruits	
*Cynara cardunculus* L. ssp*. cardunculus*	0.05	Asteraceae	stems	cardone, scardunecch’	boiled, pan-fried	
*Diplotaxis tenuifolia* (L.) DC	0.12	Brassicaceae	leaves		salad, pasta topping, pizza topping	
*Equisetum arvense* L.	0.02	Equisetaceae	shoots	stoccagnung	omelettes, pan-fried	
*Ficus carica* L.	0.02	Moraceae	fruits	fic’	fresh fruits	
*Foeniculum vulgare* Mill.	0.60	Apiaceae	seeds (I),leaves (II), stems (III)	finuch’	flavouring (I, II), liqueurs (I), mixed vegetables soup (II, III) also with beans	improves digestion (I)
*Fragaria vesca* L.	0.19	Rosaceae	fruits		fresh fruits	
*Glycyrrhiza glabra* L.	0.21	Fabaceae	roots (I), leaves and stem (II)	rrarc	rural snack (I)	feet sweating (II)
*Humulus lupulus* L.	0.19	Cannabaceae	shoots	gupl’	fried with eggs and salami, soup	
*Lactuca serriola* L.	0.10	Asteraceae	leaves	scarola	salad	
*Lactuca virosa* L.	0.03	Asteraceae	leaves	scarola	salad, soup	
*Laurus nobilis* L.	0.14	Lauraceae	leaves	laur’	flavouring	
*Leopoldia comosa* (L.) Parl.	0.47	Asparagaceae	bulbs	cipullun’,	pickle, pan-fried	soothing of burning eyes (rubbing on the temples)
*Malus sylvestris* Mill.	0.02	Rosaceae	fruits	pomo silvestr’	fresh fruits	
*Mentha pulegium* L.	0.14	Lamiaceae	leaves	piliesc’	flavouring	
*Mentha spicata* L.	0.14	Lamiaceae	leaves		flavouring	
*Mespilus germanica* L.	0.03	Rosaceae	fruits	nespulë	fresh fruits	
*Morus alba* L.	0.03	Moraceae	fruits	cieusi	fresh fruits	
*Origanum vulgare* L.	0.19	Lamiaceae	leaves	arigan’	flavouring	
*Papaver rhoeas* L.	0.29	Papaveraceae	leaves	paparina	mixed vegetable soup	
*Pastinaca sativa* L.	0.22	Apiaceae	roots	rrarc pastanacc’	with eggs, buttered and fried	
*Portulaca oleracea* L.	0.31	Portulaceae	leaves	purchiazz’	salad	
*Picris hieracioides* L.	0.17	Asteraceae	leaves	spruin’	mixed vegetable soup	
*Prunus spinosa* L.	0.07	Rosaceae	fruits		fresh fruits	
*Pyrus pyraster* (L). Du Roi	0.07	Rosaceae	fruits		fresh fruits	
*Robinia pseudoacacia* L.	0.09	Fabaceae	flowers	cagg’	rural snack, pancakes	
*Rosmarinus officinalis* L.	0.07	Lamiaceae	leaves		flavouring	
*Rubus* spp.	0.22	Rosaceae	fruits	rivital’	fresh fruits	
*Ruscus aculeatus* L.	0.10	Asparagaceae	shoots		fried with eggs and salami	
*Sambucus nigra* L.	0.10	Adoxaceae	flowers		liqueur, omelette, pancake	stomach ache (decoction together with chamomile)
*Silybum marianum* (L.) Gaertn.	0.05	Asteraceae	stems	cardone, scardunecch’	boiled	
*Sinapis alba* L.	0.03	Brassicaceae	leaves	sinap’	mixed vegetable soup, pan-fried	
*Sinapis arvensis* L.	0.38	Brassicaceae	leaves	ass’n	mixed vegetable soup, pan-fried	
*Sonchus* spp. (*oleraceus* L., *asper* L., *arvensis* L.)	0.76	Asteraceae	leaves	sivon’	mixed vegetable soup, pan-fried	
*Sorbus domestica* L.	0.10	Rosaceae	fruits	sur’v	fresh fruits	
*Onopordum acanthium* L.	0.03	Asteraceae	stems	cardone, scardunecch’	boiled	
*Taraxacum officinalis* Weber	0.09	Asteraceae	leaves	pasc’ percor’	mixed vegetable soup, pan-fried	
*Urtica* spp. (*dioica* L., *urens* L.)	0.29	Urticaceae	leaves	lurdicul’	mixed vegetable soup, omelette	
*Ziziphus jujuba* Mill.	0.05	Rhamnaceae	fruits	scesc’l	fresh fruits	

Roman numbers indicate the correlation between the traditional culinary use and a specific part of the plant

*RFC* Relative Frequency of Citation Index

Medicinal use: in brackets the way the plants are prepared and administered to give the mentioned therapeutic effect

The ethnobotanical RFC index indicates, for a given folk species and analysed area, the degree of knowledge shared among the informants. It may vary from 0 to 1, consequently, a RFC value close to 1 means that a species is very important from a cultural and traditional point of view. Overall, the best known and most consumed wild food species is *Cichorium intybus* (RFC 0.95), followed by *Sonchus* spp. (*S. oleraceus* L., *S. asper* L., *S. arvensis* L.) (RFC 0.76), *Armoracia rusticana* Gaertn., Mey. et Scherb. (RFC 0.68), *Foeniculum vulgare* (RFC 0.60) and *Beta vulgaris* L. ssp.*maritima* (L.) Arcang. (RFC 0.52) (Table 1).

Most of the recorded species, such as *C. intybus*, *Sonchus* spp*.*, *F. vulgare*, *Borago officinalis*, *Papaver rhoeas* L., *B. vulgaris* [4, 9, 13, 17, 24] (Table 1), are commonly used southern Italy and other Mediterranean areas, both for food and medicinal purposes.

The ethnobotanical research on wild food plants traditionally consumed in the Basilicata region dates back to the late 90s of the last century [25] with the areas close to the city of Potenza, in the north of the region, being the most studied. This research pointed out 230 plants used for food or aromatic purposes.

Other studies regarding wild food plants were carried out in the Tyrrhenian sector of Basilicata [17] and among the Arbëreshë people (based in northern Basilicata) that came to the region as immigrants during the XV and XVI centuries as a consequence of the Turkish invasion of Albania, and since then have lived there as quite a close community [13, 15]. In particular, among wild and weedy plants known and present in the territory, the Arbëreshë women clearly distinguish between *liakra* (edible weedy vegetables with also medicinal use) and *bara* (non-edible grasses and herbs). The term *liakra* is used as a synonym for ‘leaves’ and is of Albanian origin [24, 26]. Many of these *liakra* species are also reported in the present survey and, in particular *C. intybus, Sonchus asper*, *Clematis vitalba* L., *Diplotaxis tenuifolia* L., are among those most cited by the informants.

**Fig. 2:**
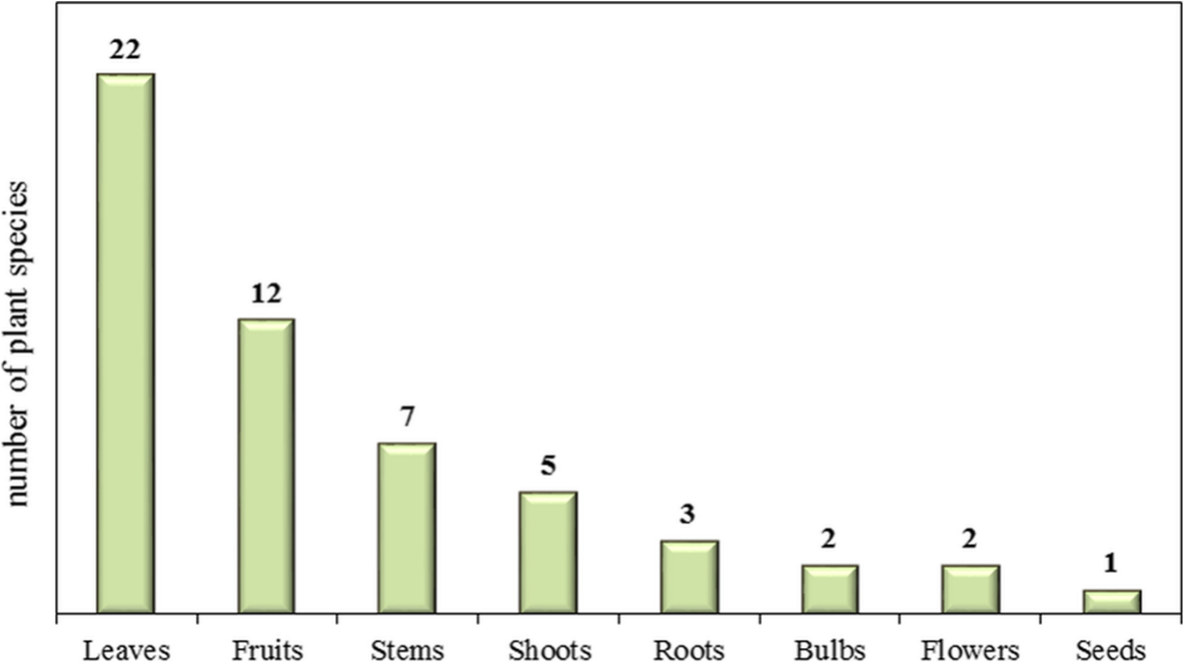
Used parts of the wild food plants traditionally consumed in the study area. The number above each bar indicates the total number of species used in each category

The folk plant species mentioned by the informants belonged to 23 different botanical families (Table 2), with Asteraceae (12 plant species, 23%) and Rosaceae (7 plant species, 13%) being the most representative (Table 2). The parts of the plants mostly used were leaves (22) and fruits (12), followed by stems (7) (Fig. 2). Five of the mentioned wild fruits are gradually becoming less common and almost not in use anymore (*Arbutus unedo* L., *Cornus mas* L., *Prunus spinosa* L., *Sorbus domestica* L., *Ziziphus jujuba* Mill.); on the contrary, other species, although only growing in specific parts of the study area, are still largely collected and appreciated (e.g., *Ficus carica* L., *Fragaria vesca* L., *Rubus ulmifolius* Scott.).

**Table 2:** Botanical families of wild food plants traditionally consumed in the Middle Agri Valley area

Botanical family	N° of wild food plant species
Asteraceae	12
Rosaceae	7
Brassicaceae	4
Lamiaceae	4
Apiaceae	3
Asparagaceae	3
Fabaceae	2
Moraceae	2
Amaranthaceae	1
Adoxaceae	1
Amaryllidaceae	1
Boraginaceae	1
Cannabaceae	1
Capparaceae	1
Cornaceae	1
Equisetaceae	1
Ericaceae	1
Lauraceae	1
Papaveraceae	1
Portulaceae	1
Ranuncolaceae	1
Rhamnaceae	1
Urticaceae	1

### Traditional foods and dishes using wild plants

Born out of necessity due to hunger, wars, drought and poverty, the culinary use of wild plants has slowly established itself in the territory, becoming part of its habits and protagonist of various traditional foods and dishes. As reported by informants, Middle Agri Valley local cuisine is poor and simple but racy, tasty and savoury.

**Fig. 3:**
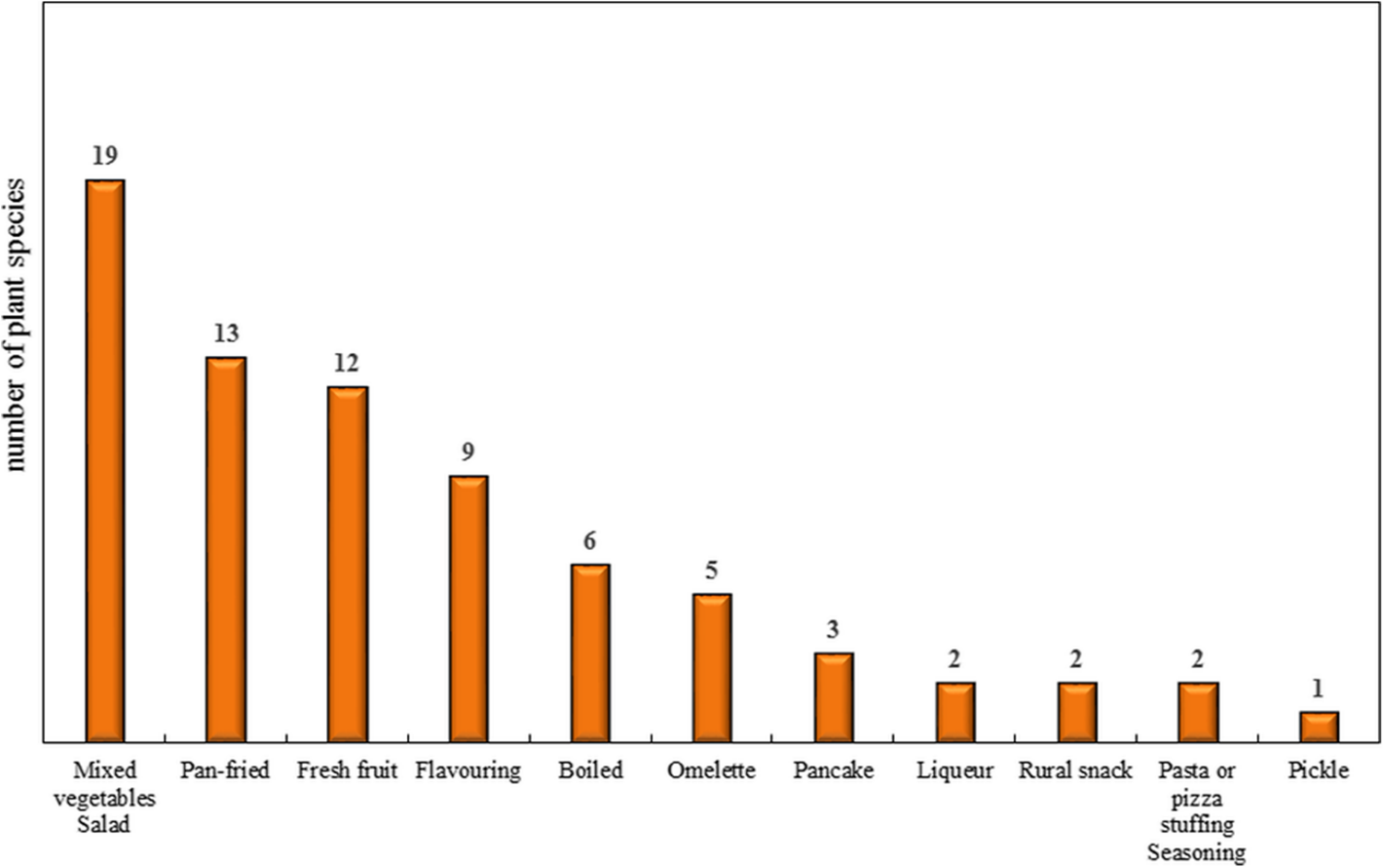
Culinary uses of the wild food plants traditionally consumed in the study area. The number above each bar indicates the total number of species used in each category

The ways of consumption of wild food plants and the number of species in each category are shown in Fig. 3. Plants are most often consumed raw, mixed with other vegetables or in salads prepared with the tender young leaves (19) collected in the early vegetative *rosetta* stage when they have a less bitter taste, or boiled, when collected as older leaves, also in mixed vegetable soups. The vegetable soups are generally prepared with beans together with *B. vulgaris*, *B. officinalis* and *C. intybus*. The wild plants are also frequently eaten pan-fried especially with eggs and local homemade salami (13), as fresh fruits (12), or added as flavouring ingredients to other preparations (9). Two species were mentioned as rural snacks: the peeled roots of *Glycyrrhiza glabra* L. and the flowers of *Robinia pseudoacacia* L., which are both sucked and particularly appreciated by children for their sweet flavour. In today’s society children have few opportunities to be in contact with nature in a free and independent way and also have ready-to-use sweet products available at their homes. For these reasons, the rural snacks mentioned by the informants were always described as something belonging to the past. Instead, wild fennel seeds (*F. vulgare*) are still commonly used in the Middle Agri Valley to aromatize salami giving them a recognizable taste. Wild asparagus spears (*A. acutifolius*) are boiled and fried with olive oil, garlic, eggs and homemade salami. Bulbs of “*lampascioni*” (*Leopoldia comosa* L. Parl.) are particularly relished, either macerated in cold water or boiled and prepared in different ways: in oil, pickled, seasoned with olive oil, garlic and chilli or fried with garlic, tomatoes and dried peppers. *B. vulgaris* is used to make stuffed pizza “*calzoni”* with raisin and a particular wild dried herb called in dialect “*piliesc*” *(Mentha pulegium* L.) is used to cook escargots.

The root of *Armoracia rusticana* (“*rafano*”) was cited by informants both as present in the wild, even tough increasingly rare, and as cultivated plant. This species is very important as traditional food of the Middle Agri Valley and largely consumed, especially during winter, because it gives dishes an intense and very hot flavour. In particular, to celebrate Carnival, a thick omelette called “*rafanata*” is prepared with grated root of “*rafano*”, pecorino cheese, eggs and parsley. “*Rafano”* is also grated on local homemade pasta.

The use of some prickly adult species (Asteraceae family, Cardueae tribe) was previously reported in other studies on Basilicata region [16, 18, 25, 27]. These plants could be considered a poverty index of the population, as they are full of thorns and require a long procedure to make them edible. Early research [25] described the alimentary use of stems of *Carduus pycnocephalus* L., *Cirsium arvense* L. Scop., *Cynara cardunculus* L. ssp. *cardunculus*, *Onopordum* L. spp. (cut into small pieces and fried); flowers and leaves of *Silybum marianum* (L.) Gaertn. (boiled or cooked in a soup), and, not recorded in this study, *Cnicus benedictus* L.*, Galactites tomentosa* Moench and *Eryngium campestre* L. (Apiaceae family) (boiled). In the present research in the Middle Agri Valley, the informants referred to the food use (boiled and/or pan-fried) of some of the above-mentioned plants only as a past habit, and they were not anymore able to distinguish among the different species indicating them all with the same folk name (“*scardunecch”*) (Table 1).

### Medicinal use of wild food plants

In the present survey, only a few wild food species were mentioned for their medicinal properties (6 species out of 45, 13%) (Table 1). The seeds of *F. vulgare* were reported to improve digestion in analogy with the only previously published ethnobotanical study in the Agri Valley area [18], which reported an infusion of these seeds together with other plants to promote the ejection of intestinal gas. Confirming their use as gastrointestinal remedy, *F. vulgare* seeds are still commonly added to bean soup in the Midlle Agri Valley. The aerial parts (leaves and stems) of *G. glabra* were mentioned to be used to prevent excess feet sweating and the bulbs of *L. comosa* rubbed on the temples to soothe burning eyes. Flowers of *Sambucus nigra* L. are used together with those of chamomile to prepare a decoction for relieving stomachache (Table 1). The cooking water of some wild greens, like *A. acutifolius* and *C. intybus*, is also used for therapeutic purposes, respectively, for the wellbeing of kidneys and liver.

Some plant species previously reported by Capasso et al. [18] as phytoremedies, such as *Laurus nobilis* and *Origanum vulgare*, were also mentioned by the informants but only to be used as food and without any relation to possible therapeutic properties, indicating the loss of such knowledge over the years. This pattern was also confirmed by comparing the present results with other studies carried out few years ago in Basilicata region both among Italians and Arbëreshë communities [13, 15, 16, 26]. In particular, many species mentioned in the present research as having only food use, were previously indicated as also showing a medicinal application such as *A. rusticana* (anti-rheumatic), *C. vitalba* (heal mouth inflammation), *C. cardunculus* (anti-rheumatic, digestive), *L. nobilis*, *F. carica*, *G glabra*, *M. domestica* and *Z. jujuba* (heal sore-throat), *Rubus* spp. and *P. spinosa* (hepato-protective), *S. marianum* (laxative), *S. oleraceus* (anti-gastritis), *P. rhoeas* (mild sedative). The most common preparation methods were decoction of aerial parts, root or fruits, or ground and topically applied. In addition, some species that have a well-known medicinal use in other parts of Italy, were not mentioned as having particular therapeutic effects in the Midlle Agri Valley. In particular, no medicinal properties were reported for *Urtica* spp., which in many other studies in Italy and abroad is known both as food and for medical use (refreshing, against kidney problems and for arthritis), and for *Taraxacum officinale* Weber and *Sonchus* spp., that have previously been defined as *medicinal foods* with both high nutritional values and depurative, blood cleaning and refreshing effects [12, 28].

### Taste of wild food plants and perceived health impacts

The bitter taste typical of many greens, in particular of plants belonging to the Asteraceae family, was often partially neutralized by boiling or by leaving the plant soaking in water for many hours. Among the listed plants (Table 1), those reported to be the most bitter were *C. intybus*, *C. vitalba*, *L. comosa* and *Ruscus aculeatus* L., with different degrees of bitterness depending on individual perception. The preference for bitter or sweet taste was very variable among the informants. Rarely a single wild green was cooked alone when making a soup, but most frequently many vegetables were mixed to reach a better final taste. *P. rhoeas* and *P. sativa,* two wild plants with a strong flavour, the latter similar to fish, are also always used in combination with other vegetables (Table 1). In general, wild food plants were perceived as healthier, more genuine and tastier than the related cultivated plant species. As a woman living in Sant’Arcangelo declared: “*In the past wild greens were eaten out of necessity, now they’re eaten for pleasure*”.

### Folk plant classification and folk names

Folk plant names were mentioned by the informants according to their own plant classification (*folk systematic*), in which the elementary unit is represented by a *folk generic*, also called *ethnospecies* [29], recognizable on the basis of differences in macro-morphology, habitat and use of the plant [30]. The present study evidenced that, in few cases, related plant species that cannot be distinguished by a non-expert were assimilated and identified as a single ethnospecies (under differentiation), in particular in the case of *Sonchus* species (e.g. *S. asper, S. arvensis and S. oleraceus*) and *Rubus* species (Table 1). It should be noted that most wild food plants are usually collected at the rosette stage or as young shoots, when the plant lacks a flower, the most important botanical identification character. During the study, difficulties were encountered when trying to link folk names to botanical names. This was mainly due to the use, in different municipalities sometimes only a few kilometres apart, of different folk names related to the same plant species; for example *C. vitalba* was called “*vitacchia”* (Aliano) and “*grambullin”* (Castronuovo, S. Andrea and Roccanova); *Sonchus* spp. was called “*sivun”* (Sant’Arcangelo), “*cardelle”* (Roccanova, San Martino) and “*iung”* (Aliano); *Sinapis arvensis* L. was called “*marogna”* (Roccanova) and “*ass’n”* (Sant’Arcangelo). None of the cited names had a meaning related to a botanical characteristic of the plant or to its use. When asked regarding the meaning of given folk names, the informants always answered that those were just the proper names of wild plants.

## Conclusions

The objective of the present study was to record the local knowledge concerning traditional wild food plants of the Middle Agri Valley area, as well as to collect information regarding the practices (gathering, processing and cooking) and therapeutic uses related to these plants. The data collected may contribute to preserve an important part of the cultural heritage still partially retained by this population. Over the last few decades, this knowledge has been quickly disappearing along with the people and, even in the rural context of the Agri Valley study area, is less and less handed down to the new generations, who choose to emigrate elsewhere in search of work and of a more modern lifestyle typical of urban areas. Nonetheless, the present survey revealed that, in spite of the loss of TLK, the use of wild plants is being revaluated today as they are perceived as healthy and represent the preservation of biodiversity as well as of old traditions and own cultural roots. In particular, in the Middle Agri Valley area, the revaluation of wild herbs seems to be closely related to local customs and traditions.

## Additional file

Additional file 1: Questionnaire form. Guidelines followed during the semi-structured interviews of the ethnobotanical survey. (PDF 78 kb)
